# Improving the Reimbursement Process for New Drugs: A Case Study of a Two‐Waiver System in South Korea

**DOI:** 10.1111/jep.70074

**Published:** 2025-04-01

**Authors:** Seung‐Rae Yu

**Affiliations:** ^1^ Dongduk Women's University Seoul Korea

**Keywords:** health economics, health policy, healthcare, public health, qualitative methods, value

## Abstract

**Objectives:**

To empirically analyse the case of a newly introduced system in Korea to improve the process of drug reimbursement to investigate the application of the waiver system to economic evaluations and pricing negotiations, to examine the characteristics and inclusion outcomes of drugs subject to this system and to derive implications for improving patient access and financial sustainability.

**Methods:**

A drug data set was compiled using data from the Ministry of Health and Welfare (MoHW) and public institutions. Additional information on pharmaceutical companies and indications was obtained from the Korea Food and Drug Administration (KFDA). Descriptive statistics were used to summarise variable distributions. Chi‐squared tests and multivariate logistic regression analyses, including interaction terms, were performed to determine differences before and after the implementation of the waiver system and their relevance to negotiation outcomes. Statistical analysis was performed using SPSS version 27.0 with a significance level of 0.05.

**Results:**

From 2007 to 2022, a total of 785 new drugs have been introduced. In Korea, the waiver system includes two types: a price negotiation waiver primarily for non‐orphan and non‐cancer drugs and an economic evaluation waiver designed for orphan and cancer drugs with significant clinical needs. Drugs listed in three or fewer countries were significantly more likely to utilise the price negotiation waiver system due to pricing disadvantages associated with limited international registration. Since the introduction of the two waiver systems, there has been a notable increase in negotiation agreement rates for orphan and cancer drugs, a trend supported by regression analysis.

**Conclusions:**

This study demonstrates how Korea's two‐waiver system streamlined the reimbursement process, improving access to orphan and cancer drugs while ensuring financial sustainability. The system's eligibility criteria effectively balance the needs of noncritical and critical diseases, ensuring equitable access while maintaining fiscal responsibility. These findings have important institutional implications for improving patient access to medicines while effectively managing financial expenditure.

## 1 Introduction

Pharmaceuticals are products that embody innovative technologies of companies. They are considered public goods with significant societal value crucially managed by the government. Pharmaceutical policies are directly linked to the health and finances of a nation. Thus, rational decision‐making is important for both patient accessibility and health insurance coverage. Consequently, evidence‐based value assessment and pricing decisions for pharmaceuticals are globally emphasised. For instance, countries like the UK, Australia and Canada have long utilised pharmacoeconomic evaluations as a vital Health Technology Assessment tool, focusing on clinical and cost benefits for individual patients who receive new drugs [1]. Additionally, pharmaceutical pricing negotiation systems utilised in Germany, France and Italy prioritise considering the total financial impact when used for the entire patient population, alongside the cost‐effectiveness for individual patients [2]. With recent acceleration in scientific and technological advancements enabling the development of new drugs for previously hard‐to‐treat diseases, expectations in the medical field have risen further. While patients criticise the inability to immediately access new drugs proven effective in clinical trials, governments face challenges in decision‐making due to substantial costs covered by public funds such as taxes and medical insurance [3]. Nonetheless, efforts to continually improve evaluation methods and procedures, particularly if therapeutic benefits exist for patients, are imperative. Despite the global emphasis on innovative drug pricing and reimbursement systems, there remains a lack of empirical studies analysing South Korea's two‐waiver system, which was introduced to improve access to innovative drugs while ensuring financial sustainability. Therefore, the aim of this study was to ascertain characteristics of the two‐waiver system implemented in South Korea in 2015, specifically their application to new drugs, and to investigate relationships between independent variables and changes observed before and after introduction of the system. This was achieved using the agreement rate of health insurance coverage negotiations as a performance indicator and dependent variable.

In this context, South Korea's two‐waiver system stands out as an innovative approach that integrates clinical and cost considerations, balancing rapid patient access with financial sustainability. This model offers valuable lessons for countries facing similar challenges in improving access to innovative drugs while maintaining budgetary controls. The core of this system lies in replacing cost‐effectiveness evaluation with health care advanced 7 countries (A7 countries) having the lowest prices for new drugs without therapeutic alternatives and substituting cost‐effectiveness evaluation with weighted average market price for drugs with therapeutic alternatives. Thus, it can be seen as an efficient institutional design as it simplifies procedures of evaluation and negotiation by reflecting both clinical and cost values of new drugs.

South Korea stands as the first country in Asia to introduce Health Technology Assessment [4], operating a unified national health insurance system and a single drug price list, ensuring high transparency and representativeness of drug prices. Particularly noteworthy is the fact that South Korea serves as a key reference market in Asia. Adjustments in its pricing and reimbursement systems can influence pharmaceutical strategies not only locally but also in other emerging economies, such as China and Saudi Arabia [5], where South Korean policies often set benchmarks for drug price negotiations and market access.

Despite this significance, empirical studies analysing South Korea's 2015 Waiver System are lacking. Since the introduction of the pharmacoeconomic evaluation system in 2007, research on the methodology and guidelines of cost‐effectiveness evaluation has been ongoing [6, 7]. However, application cases for drugs where cost‐effectiveness evaluation is infeasible have not been reported yet. Furthermore, while risk‐sharing agreement systems, such as postdrug‐cost reconciliation methods, have been researched for high‐cost severe diseases such as rare diseases and anticancer drugs [8, 9, 10], there remains a lack of empirical studies from the perspective of patient accessibility regarding many new drugs for chronic diseases that are not subject to risk‐sharing agreement systems.

### 1.1 Korean Healthcare System and Drug Pricing System

The healthcare system in South Korea operates within the National Health Insurance (NHI) system, ensuring that all citizens are enroled in a unified health insurance program. Under this system, healthcare institutions and pharmaceutical companies play important roles as users and providers of medical products within the scope of health insurance coverage [11].

For patients to access new medications, they must be included in the scope of health insurance coverage. Drug prices are ultimately determined through negotiations between the government and pharmaceutical companies [12]. The Health Insurance Review and Assessment Service (HIRA) conducts evaluations of clinical utility and cost‐effectiveness before drug pricing negotiations, which is a crucial aspect. However, meeting HIRA's evaluation criteria does not guarantee inclusion in the National Health Insurance Drug List. The final decision on drug inclusion depends on mutual agreement through drug pricing negotiations supervised by the National Health Insurance Service (NHIS) [13, 14].

In 2007, South Korea introduced the ‘Drug Cost Rationalization Plan’, which aimed to apply health insurance only to new drugs with clinical and economic value [15]. Also known as the ‘positive list’ system, pharmaceutical companies submit registration applications to the MoHW. Subsequently, the HIRA, an affiliate of the MoHW, evaluates the appropriateness of coverage and the NHIS negotiates the final price [16].

### 1.2 Introduction of the Waiver System

In the operation process of the ‘Positive List’ system as described above, practical limitations have been revealed. Firstly, in cases of rare diseases or terminal cancer drugs where the number of patients is small, it is difficult to obtain statistically significant clinical and cost‐related evidence. Despite these challenges, the HIRA mandates pharmacoeconomic evaluations, leading to issues with therapeutic accessibility. Additionally, in cases of mild chronic diseases where numerous alternative drugs have already existed, HIRA's evaluation process has already calculated economically viable prices. However, despite this, compulsory additional price negotiations with the NHIS have led to unnecessary administrative efforts and time consumption. For these reasons, the ‘Positive List’ system needed further development based on drug and therapeutic applications, prompting South Korea to enact institutional changes in 2015.

#### 1.2.1 Waiver System of Drug Price Negotiation

The first change was the exemption from drug price negotiations. This system can be applied when there are alternative drugs equivalent to the new drug in terms of clinical status. Under this system, the price of the new drug is set at approximately 90% of the weighted average price (WAP) of alternative drugs. However, there are some exceptions. For example, orphan drugs and biologics can be priced at 100% and those with pediatric indications can be priced at 95%. In Korea, at the time of listing a new drug, the NHIS and pharmaceutical companies agree on the expected revenue, based on which a Price–Volume Agreement (PVA) system for drug price post‐management is implemented. While the time and procedures required for drug price negotiations have been simplified compared to the past, negotiations regarding expected revenue remain crucial. If an agreement on expected revenue cannot be reached, the new drug cannot be listed under health insurance.

#### 1.2.2 Waiver System of Pharmacoeconomic Evaluation

The second change was the exemption from pharmacoeconomic evaluation. This system applies when there are no alternative drugs equivalent to the new drug in terms of clinical status. Additionally, it is only applicable when the number of patients is extremely small, specifically fewer than 200. This policy decision was based on historical data from South Korea, where the upper limit for patients with extremely rare diseases covered by insurance was used as a reference. Despite challenges in generating significant evidence due to a small patient population, criteria for cost‐effectiveness assessment are still necessary. Therefore, instead of conducting a traditional pharmacoeconomic evaluation, HIRA utilises the method of External Reference Pricing (ERP), referencing prices in A7 countries (United States, Japan, United Kingdom, France, Germany, Italy and Switzerland), which represent high‐income markets with advanced healthcare systems. This ensures that the prices set in South Korea are aligned with global standards for cost‐effectiveness and affordability. Specifically, cost‐effectiveness is recognised if the drug price is below the minimum price in these A7 countries. Subsequently, during negotiations with the NHIS, drug prices are negotiated by referencing prices from OECD countries other than A7 countries. The agreement on the appropriate expected revenue after listing is also reached through negotiations. Since these drugs are often the only option for patients, the final negotiation agreement rate under this system is considered a crucial indicator of treatment accessibility.

## 2 Methods

As mentioned in the introduction, Korea's 2015 two‐waiver system aimed to adhere to the principles of the positive list while simultaneously enhancing patient access to treatment [17]. Therefore, the design and key assumptions of this study were: (1) there would be differences in distributional trends of products that passed the HIRA evaluation and reached NHIS negotiations as of 2015, the year the two‐waiver system was introduced and (2) successful negotiation outcomes leading to health insurance coverage would be associated with characteristics of each product. Based on investigation results of each variable, a data set was constructed and basic status and trends were confirmed through descriptive statistics. Subsequently, negotiation outcomes between NHIS and pharmaceutical companies were set as dependent variables and the degree and directionality of significant associations among remaining independent variables were statistically tested. To construct the data set, information on new drugs evaluated by HIRA and negotiated by NHIS from 2007 to 2022 was thoroughly examined. Analysis variables were classified as follows:

1. Company type: Companies were classified into domestic and multinational companies based on locations of their headquarters.
2. Drug characteristics: Considering the severity of the disease and the necessity of patient accessibility, drugs designated as orphan drugs by the KFDA and drugs indicated for cancer were distinguished.
3. Negotiation result: Negotiations conducted by the NHIS and pharmaceutical companies were classified into whether they ultimately reached agreement or ended in disagreement. Negotiation result served as a dependent variable and an indicator of the system's performance in this study. It was subjected to statistical testing of causality through regression analysis.
4. PVA history: PVA history was investigated as a financial post‐management indicator of new drugs. In Korea, the PVA system operates by reducing drug prices if actual sales volume exceeds annually agreed‐upon expected sales volume by more than 30% after the introduction of the new drug [18]. Therefore, if a new drug is subject to PVA after its introduction, it can be interpreted as a case where the financial burden is greater than initially anticipated.
5. ERP country: As an indicator considered in NHIS negotiations, the classification of whether a new drug has been registered in three or fewer foreign countries was examined and established as an ERP evidence variable. This is necessary because, according to the NHIS Drug Price Negotiation Guidelines for New Drugs [19], new drugs registered in three or fewer foreign countries are subject to a significant penalty in pricing (specifically, a price at least 20% lower than the reference price applicable to general new drugs). Consequently, for drugs lacking ERP evidence, negotiating prices with the NHIS can be highly disadvantageous from a market perspective. Therefore, it is important to classify new drugs based on ERP evidence and examine their distributional characteristics and their association with negotiation outcomes before and after the introduction of the Waiver System.

### 2.1 Data Collection and Data Sources

To gather information on new drugs and pricing negotiation in Korea, publicly available data from government agencies were utilised. In South Korea, the MoHW publicly discloses information on how drugs are covered by health insurance, including specific prices and coverage details, ensuring accessibility for all citizens. The HIRA and the NHIS, both under MoHW, make detailed information on the evaluation and negotiation processes of health insurance drugs, as well as lists of these drugs, available through their respective websites. Additionally, the Ministry of Food and Drug Safety (MFDS) and pharmaceutical associations offer information on drug approval and supplier details via their websites. These resources are publicly accessible, and therefore, there was no need to obtain special permission or access rights for this study. The following declaration outlines the specific sources and methods used for data collection in this study.

The HIRA provided the Health Insurance Drug Benefit List [20] and the Pharmaceutical Reimbursement Evaluation Results Disclosure [21]. Additionally, the NHIS provided a list of negotiated medicines for drug pricing [22]. These sources were used to complete the data set with basic product information and pricing negotiation‐related data.

Based on this foundation, information on whether a drug is classified as an orphan drug or an anticancer agent was obtained through the MFDS Pharmaceutical Approval Information Database [23]. Moreover, data disclosed by the Korea Research‐based Pharmaceutical Industry Association (KRPIA) and the Korea Pharmaceutical and Bio‐Pharma Manufacturers Association (KPBMA) were used to classify pharmaceutical company characteristics [24, 25].

Furthermore, the NHIS foreign drug price reference sites [23] were used to ascertain the ERP status of a drug under investigation. Finally, the MoHW's Committee prior information announcement [24] was consulted to investigate whether drug prices were reduced through the PVA system after a drug was listed. To ensure consistency and accuracy, data from multiple public sources were cross‐referenced. Publicly disclosed information on drug prices and reimbursement details was matched with HIRA and NHIS records to ensure alignment. Any discrepancies among sources were addressed through additional verification using public records from the MoHW, as well as pharmaceutical approval information from MFDS.

### 2.2 Statistical Analyses

For the analysis of collected data, SPSS version 27.0 (IBM SPSS Statistics for Windows, Armonk, NY: IBM Corp; 2020) was utilised. Main findings were summarised using descriptive statistics. This study utilised a full census of drugs subject to the waiver system, ensuring that findings are based on the entire population rather than a sampled subset. As a result, traditional power analysis and sample size justification were not applicable. To examine trends before and after implementation of the Waiver System and identify factors influencing overall negotiation outcomes for new drugs, Chi‐square tests and multivariable logistic regression analyses were conducted at a significance level of 0.05.

Logistic regression analysis employed a backward elimination algorithm known for minimising the negative impact of correlations between explanatory variables [25]. The goodness of fit of a regression model was assessed using the Hosmer–Lemeshow method [26, 27]. Additionally, the Nagelkerke *R* ^2^ coefficient was provided as a measure of how much variation in a dependent variable could be explained by the regression model [28, 29]. The −2 Log likelihood statistic was also presented as an indicator of model fit, utilising the maximum likelihood estimation method [30, 31]. Additionally, interaction effects between key variables, such as ERP status and PVA application, were explored to assess their combined impact on negotiation outcomes [32, 33]. These interaction terms provided additional insights into the dynamics of the waiver system's implementation.

## 3 Results

### 3.1 Overview Analysis

From 2007 to 2022, a total of 785 new drug products underwent negotiations. Excluding the initial year of implementation in 2007 (*n* = 10), negotiations were conducted for an average of 49 products per year (Table 1).

**Figure 1:**
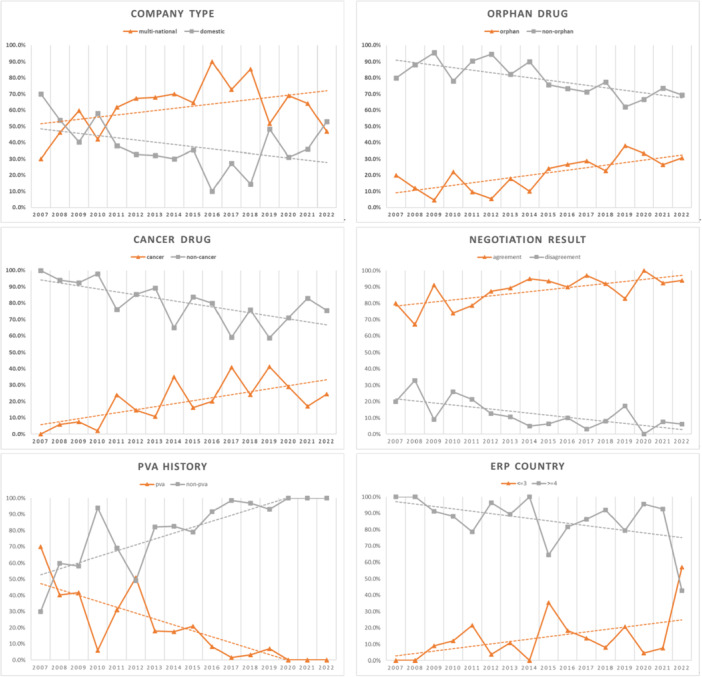
Weighted values for each analytic variable by year.

The distribution and proportion of negotiations by product characteristics (analytical variables) were compared by year as shown in Figure 1. Firstly, regarding company characteristics, multinational companies (*n* = 503) outnumbered domestic companies (*n* = 282), with their proportion increasing steadily from 2007 to present. In terms of disease characteristics, numbers of orphan drugs (*n* = 159) and cancer drugs (*n* = 155) were lower than those of non‐orphan and non‐cancer drugs, respectively. However, their relative proportions showed an increasing trend over time. The average negotiation agreement rate for all the drugs was approximately 88.2%, showing a continuous increase over time. Products subject to the PVA system (*n* = 141) accounted for around 18% of the total (*n* = 785), with their proportion steadily decreasing. Lastly, regarding the ERP basis variable, products listed in three or fewer foreign countries (*n* = 113) accounted for approximately 14% of the total (*n* = 785), with their proportion steadily increasing over time.

### 3.2 Comparison Analysis Before and After the Waiver System

Based on findings from Section 3.1, a comparison was made between periods before and after the introduction of the Waiver System in 2015 (Table 2). A notable change observed was significant increases in proportions of orphan drugs (11.1% → 27.9%) and cancer drugs (12.5% → 25.8%) after 2015 compared to before. The number of drugs that were both cancer drugs and orphan drugs was very small, totaling 61, which constituted only about 7.7% of the entire list of drugs (*n* = 785). Among these, a mere seven drugs were exempted from price negotiations, which was relatively rare. However, 33 of these drugs were granted exemptions from pharmacoeconomic evaluation, representing a relatively large proportion of the total pharmacoeconomic evaluation exemptions (*n* = 53) granted since 2015. Additionally, the proportion of drugs listed in three or fewer foreign countries serving as the baseline for price penalties under the ERP basis increased nearly three‐fold (7.2% → 20.4%). In contrast, the proportion of drugs subject to the PVA system after listing showed a substantial decrease (32.9% → 5.4%). These differences were statistically significant for all the variables (all *p* < 0.001).

### 3.3 Analysis of Factors Related to the Waiver System

To examine differences between the two types of Waiver Systems based on product characteristics, we classified them accordingly (Table 3). Firstly, products exempt from pricing negotiation showed statistically significant differences in all the variables except for company type. Specifically, these products had significantly higher proportions of non‐orphan and non‐cancer drugs as well as a higher negotiation agreement rate. Notably, the proportion of products listed in three or fewer foreign countries was over seven times higher in products exempt from pricing negotiation (36.8%) compared to that of products undergoing pricing negotiation (5.1%). Conversely, regarding the application of the PVA system, the proportion of products exempt from pricing negotiation (2.2%) was significantly lower than that of products undergoing pricing negotiation (24.5%).

For products exempt from pharmacoeconomic evaluation (Table 4), statistically significant differences were observed in all the variables except for negotiation agreement rate. Unlike products exempt from pricing negotiation, the majority of products exempt from pharmacoeconomic evaluation had higher proportions of orphan drugs and cancer drugs. Additionally, among products exempt from pharmacoeconomic evaluation, no cases were found where they were listed in three or fewer foreign countries. No cases were found where the PVA system was applied either.

### 3.4 Factor Analysis of Negotiation Agreement Rate and Other Variables

Variables that showed significant differences in negotiation outcomes were period, ERP basis, and exemption from pricing negotiation (Table 4). For other variables such as multinational pharmaceutical companies, non‐orphan drugs and non‐anticancer drugs, the negotiation agreement rate was relatively higher but statistically insignificant. The negotiation agreement rate after 2015 was approximately 11.0 percentage points higher compared to that before 2015, which was statistically significant. Negotiation agreement rates for products listed in three or fewer foreign countries and products exempt from pricing negotiation were 8.7 percentage points and 12.5 percentage points higher, respectively, compared to those that did not fall into these categories.

Furthermore, subgroup analysis revealed that among 231 products exempt from pricing negotiation, 85 were listed in three or fewer foreign countries, accounting for approximately 75.2% of the total. In contrast, among 554 products where pricing negotiation was not exempted, only 28 were listed in three or fewer foreign countries, comprising approximately 24.8% of the total. Therefore, for products listed in three or fewer foreign countries, the proportion of products covered by health insurance through the exemption from pricing negotiation introduced after 2015 has increased.

### 3.5 Regression Analysis Related to Negotiation Agreement Rate

To understand the ultimate impact of product and company characteristics as well as the introduction of the Waiver System on the health insurance listing of new drugs, a multivariable logistic regression analysis was conducted with negotiation outcome as the dependent variable (Table 5). In both Model 1 where all variables were included and Model 2 that performed analyses using the Backward elimination method, period, orphan drug and pricing exemption variables were found to have significant effects, consistent with trends observed in the descriptive statistics.

Specifically, the period after 2015 and the exemption from pricing negotiation positively influenced the negotiation agreement rate, while the orphan drug variable had a relatively negative impact on the negotiation agreement rate. Additionally, considering that the distribution of independent variables might be influenced by the introduction of the Waiver System (2015), interactions between the period variable and other independent variables were included in the regression analysis in Model 3.

Results revealed that the cancer drug variable also had a negative impact on the negotiation agreement rate, like orphan drug variable in terms of drug price and financial impact. However, when considering interactions with the period variable, both orphan drugs and anticancer agents negotiated after 2015 had a positive impact on the negotiation agreement rate compared to before 2015.

## 4 Discussion

This study is significant for analysing comprehensive records of pharmaceutical price negotiations in South Korea from 2007 to 2022, identifying trends, and exploring associations and causality among key variables. This study acknowledges that temporal confounders, such as policy changes, economic conditions and shifts in the pharmaceutical industry, could have influenced negotiation outcomes. These external factors were not explicitly accounted for in the analysis but may have played a role in observed trends, such as the increasing agreement rate over time. For instance, changes in government reimbursement policies or fluctuations in global pharmaceutical market dynamics could have impacted both company strategies and negotiation processes. Future studies should consider these contextual elements to provide a more nuanced understanding of negotiation dynamics.

Similar waiver systems have been reported internationally. For example, in Australia and New Zealand, life‐threatening or rare disease treatments may be exempt from economic evaluations and listed through separate funding mechanisms [34, 35]. In Germany and France, treatments demonstrating therapeutic efficacy may bypass price negotiations, enabling swift listing at lower costs [36, 37]. By analysing South Korea's two‐waiver system over an extended period (2007–2022), this study provides empirical insights into the impact of these significant policy changes on drug listing. This comparative perspective enhances the understanding of how Korea's approach aligns with global practices and its implications for drug access and policy effectiveness.

With the advancement of science and technology worldwide, there is a growing trend towards the development of innovative drugs, especially for niche markets such as rare diseases and cancer treatments. It is primarily driven by major multinational pharmaceutical companies. Consequently, South Korea has observed a continual increase in the negotiation share of products defined by such variables in new drug negotiations. Interpretation of negotiation agreement rates suggests an overall increase over time attributed to the accumulation of negotiation experience and improved mutual understanding and acceptance between the government and pharmaceutical companies. However, the decreasing trend in PVA linked to fiscal expenditure after each year's new drug negotiation implies potential overestimation of expected revenues set by the government and pharmaceutical companies’ initial registration or lower‐than‐expected actual revenues post‐registration. This highlights the necessity to address uncertainties in future fiscal management systems. Moreover, the notable observation of a higher proportion of products registered in fewer than three countries being particularly prevalent in the pricing exemption system warrants attention. The increasing utilisation of the pricing exemption system by relatively less internationally experienced new drugs could lead to increased clinical and cost uncertainties from the government's perspective. Comparing before and after the introduction of the Waiver System in 2015, proportions of products from multinational pharmaceutical companies, rare disease drugs and anticancer drugs have shown a comprehensive increase. The pharmacoeconomic evaluation exemption system is mainly applied to rare disease drugs and anticancer drugs, while the pricing exemption system is more prevalent for non‐orphan drugs and non‐anticancer drugs, indicating complementary operation between the two systems. Although regression analysis identified variables influencing negotiation agreement rates, it was challenging to confirm a model with sufficient explanatory power. The Nagelkerke *R* ^2^ value, which is commonly used as a measure of explanatory power, reached the highest value of 0.116 in Model 3, falling short of the typical range (0.2 – 0.4) for indicating an adequate model fit [38]. This limitation highlights the inherent complexity of the negotiation process, which involves multilevel decision‐making and qualitative factors that are difficult to quantify. The inclusion of interaction terms improved the explanatory power slightly, suggesting that the combined effects of variables are critical to understanding negotiation outcomes. For example, ERP penalties and PVA application may jointly influence the financial risks associated with new drugs, highlighting the need for an integrated policy framework to address their combined impact. Exploring such interactions could further clarify the financial and clinical implications of these exemptions. However, the negotiation process remains subject to multiple influencing factors, including external and contextual elements, that were not fully captured in the model [39]. Future studies should refine variable categorisations or include additional predictors to address these challenges and enhance model performance. Despite the general negative impact of rare disease drugs and anticancer drugs on negotiation agreement rates, the positive impact observed after the introduction of the Waiver System in 2015 highlights the effectiveness of policies prioritising rapid access to essential treatments. The differential effects of the pricing and pharmacoeconomic evaluation exemption systems also indicate their complementary roles: pricing exemptions are more commonly applied to non‐orphan, non‐cancer drugs, while pharmacoeconomic evaluation exemptions are primarily targeted at rare diseases and cancer treatments. These distinctions suggest that policy adjustments could further optimise the balance between accessibility and financial sustainability. Furthermore, it should be noted that the waiver of drug pricing negotiations does not guarantee a 100% negotiation agreement rate for all the products. Negotiations between pharmaceutical companies and the NHIS regarding projected sales figures are also crucial. Adequate projected sales figures must be negotiated to ensure a rational operation of the PVA system in the future.

**Table 1:** Number of new drug negotiations by analysis variable.

Variables		2007	2008	2009	2010	2011	2012	2013	2014	2015	2016	2017	2018	2019	2020	2021	2022	Total
Company type	Multi‐national	3	31	40	21	26	37	19	28	40	54	48	53	15	31	34	23	503
	Domestic	7	36	27	29	16	18	9	12	22	6	18	9	14	14	19	26	282
Orphan drug	Orphan	2	8	3	11	4	3	5	4	15	16	19	14	11	15	14	15	159
	Non‐orphan	8	59	64	39	38	52	23	36	47	44	47	48	18	30	39	34	626
Cancer drug	Cancer		4	5	1	10	8	3	14	10	12	27	15	12	13	9	12	155
	Non‐cancer	10	63	62	49	32	47	25	26	52	48	39	47	17	32	44	37	630
Negotiation result	Agreement	8	45	61	37	33	48	25	38	58	54	64	57	24	45	49	46	692
	Dis‐agreement	2	22	6	13	9	7	3	2	4	6	2	5	5	—	4	3	93
PVA history	PVA	7	27	28	3	13	28	5	7	13	5	1	2	2	—	—	—	141
	Non‐PVA	3	40	39	47	29	27	23	33	49	55	65	60	27	45	53	49	644
ERP country	≤ 3			6	6	9	2	3	—	22	11	9	5	6	2	4	28	113
	≥ 4	10	67	61	44	33	53	25	40	40	49	57	57	23	43	49	21	672

**Table 2:** Distribution comparison before and after the two‐waiver system.

Variables		Before 2015		After 2015		Total	
		Product	Portion	Product	Portion		
Company type***	Multi‐national	205	57.1%	298	70.0%	503	64.1%
	Domestic	154	42.9%	128	30.0%	282	35.9%
Orphan drug***	Orphan	40	11.1%	119	27.9%	159	20.3%
	Non‐orphan	319	88.9%	307	72.1%	626	79.7%
Cancer drug***	Cancer	45	12.5%	110	25.8%	155	19.7%
	Non‐cancer	314	87.5%	316	74.2%	630	80.3%
Negotiation result***	Agreement	295	82.2%	397	93.2%	692	88.2%
	Disagreement	64	17.8%	29	6.8%	93	11.8%
PVA history***	PVA	118	32.9%	23	5.4%	141	18.0%
	Non‐PVA	241	67.1%	403	94.6%	644	82.0%
ERP country***	≤ 3	26	7.2%	87	20.4%	113	14.4%
	≥ 4	333	92.8%	339	79.6%	672	85.6%
Total		359	100%	426	100%	785	100%

*** *p* < 0.001.

**Table 3:** Comparison of characteristics of products in a waiver system.

Variables		Waiver system of price negotiation					Waiver system of pharmacoeconomic evaluation				
		Non‐waiver		Waiver		*p*‐value	Non‐waiver		Waiver		*p*‐value
		Product	Portion	Product	Portion		Product	Portion	Product	Portion	
Company type	Multi‐national	148	64.1%	355	64.1%	0.998	43	81.1%	460	62.8%	0.007
	Domestic	83	35.9%	199	35.9%		10	18.9%	272	37.2%	
Orphan drug	Orphan	34	14.7%	125	22.6%	0.013	46	86.8%	113	15.4%	< 0.001
	Non‐orphan	197	85.3%	429	77.4%		7	13.2%	619	84.6%	
Cancer drug	Cancer	20	8.7%	135	19.7%	< 0.001	39	73.6%	116	15.8%	< 0.001
	Non‐cancer	211	91.3%	419	80.3%		14	26.4%	616	84.2%	
Negotiation result	Agreement	224	97.0%	468	84.5%	< 0.001	48	90.6%	644	88.0%	0.573
	Disagreement	7	3.0%	86	15.5%		5	9.4%	88	12.0%	
PVA history	PVA	5	2.2%	136	24.5%	< 0.001	0	0.0%	141	19.3%	< 0.001
	Non‐PVA	226	97.8%	418	75.5%		53	100.0%	591	80.7%	
ERP country	≤ 3	85	36.8%	28	5.1%	< 0.001	0	0.0%	113	15.4%	0.002
	≥ 4	146	63.2%	526	94.9%		53	100.0%	619	84.6%	
Total		426	100%	359	100%		426	100%	359	100%	

**Table 4:** Comparison of negotiation agreement rates based on product characteristics.

Variables		Negotiation agreement		Negotiation disagreement		Total	
		Product	Portion	Product	Portion	Product	Portion
Period***	Before 2015	295	82.2%	64	17.8%	359	100%
	After 2015	397	93.2%	29	6.8%	426	100%
Company type	Multi‐national	447	88.9%	56	11.1%	503	100%
	Domestic	245	86.9%	37	13.1%	282	100%
Orphan drug	Orphan	134	84.2%	25	15.7%	159	100%
	Non‐orphan	558	89.1%	68	10.9%	626	100%
Cancer drug	Cancer	132	85.2%	23	14.8%	155	100%
	Non‐cancer	560	88.9%	70	11.1%	630	100%
ERP country**	≤ 3	108	95.6%	5	4.4%	113	100%
	≥ 4	584	86.9%	88	13.1%	672	100%
Waiver of PN***	Waiver	224	97.0%	7	3.0%	231	100%
	Non‐waiver	468	84.5%	86	15.5%	554	100%
Waiver of PE	Waiver	48	90.6%	5	9.4%	53	100%
	Non‐waiver	644	88.0%	88	12.0%	732	100%
Total		692	88.2%	93	11.8%	785	100%

Abbreviations: PE, pharmacoeconomic evaluation; PN, price negotiation.

** *p* < 0.01

*** *p* < 0.001.

**Table 5:** Regression analysis of factors associated with drug negotiation agreement rate.

	Model 1 (full model)				Model 2 (backyard elimination, without interaction)				Model 3 (backyard elimination, adding interaction)			
Factors	*B*	SE	Exp (*B*)	*p*‐value	*B*	SE	Exp (*B*)	*p*‐value	*B*	SE	Exp (*B*)	*p*‐value
Intercept	1.5	0.2	4.5	< 0.001	1.6	0.1	4.99	< 0.001	1.8	0.1	6.23	< 0.001
Period												
Before 2015	Ref.				Ref.				
After 2015	0.7	0.3	1.94 (1.05–3.58)	0.034	0.8	0.3	2.13 (1.21–3.76)	0.009	
Company type												
Domestic	Ref.					
Multinational	0.2	0.3	1.27 (0.77–2.09)	0.344		
Orphan drug												
Non‐orphan	Ref.				Ref.				Ref.			
Orphan	−0.6	0.3	0.53 (0.30–0.95)	0.033	−0.6	0.3	0.54 (0.31–0.94)	0.029	−1.1	0.4	0.33 (0.16–0.66)	0.002
Cancer drug												
Non‐cancer	Ref.								Ref.			
Cancer	−0.4	0.3	0.69 (0.38–1.27)	0.232					−0.8	0.4	0.47 (0.23–0.96)	0.038
ERP country												
≥ 4	Ref.					
≤ 3	0.5	0.5	1.61 (0.59–4.41)	0.355		
Waiver system of price negotiation												
Non‐waiver	Ref.				Ref.				Ref.			
Waiver	1.2	0.5	3.18 (1.22–8.30)	0.018	1.2	0.5	3.37 (1.38–8.24)	0.008	1.6	0.4	4.95 (2.22–11.00)	< 0.001
Waiver system of pharmacoeconomic evaluation												
Non‐waiver	Ref.								
Waiver	0.7	0.6	2.09 (0.69–6.31)	0.191					
Orphan drug by period												
Orphan drug, before 2015									Ref.			
Orphan drug, after 2015									1.2	0.5	3.28 (1.16–9.25)	0.025
Cancer drug by period												
Cancer drug, before 2015									Ref.			
Cancer drug, after 2015									1.1	0.5	3.11 (1.07–8.99)	0.036
Nagelkerke *R* ^2^	0.104				0.094				0.116			
−2 Log likelihood	528.089				532.184				522.515			
Hosmer–Lemeshow goodness‐of‐fit	*χ* ^2^ = 11.27, df = 8, *p* = 0.187				*χ* ^2^ = 5.656, df = 4, *p* = 0.226				*χ* ^2^ = 0.575, df = 4, *p* = 0.966			

Finally, this study has several limitations that require careful interpretation. Firstly, there was uncertainty in confirming the exact foreign inclusion status, including products exempt from drug pricing negotiations. Although this study relied on publicly available HIRA evaluations and NHIS disclosures to enhance credibility, these sources may not fully capture all the nuances of foreign inclusion or company‐specific pricing strategies. Secondly, for products where the PVA system has not been applied post‐inclusion, this study could not determine the specific reasons due to the lack of access to proprietary company data. Publicly available government data provided a general overview, but detailed comparisons between negotiated projected sales figures and actual post‐inclusion sales figures were not feasible. Such comparisons could enable a gap analysis to identify product characteristics or Waiver System factors contributing to discrepancies. If a comparison could be made between negotiated projected sales figures and actual sales figures post‐inclusion for each product, a gap analysis could be conducted to examine which product characteristics and aspects of the Waiver System contributed to the widening gap. Thirdly, while regression analysis identified key factors influencing negotiation agreement rates, it is likely that unobserved factors—such as pharmaceutical companies’ internal strategies, market‐entry intentions, or resource allocations – played a role in the outcomes [40]. These elements were beyond the scope of the available data set and may offer deeper insights in future research where proprietary or qualitative data can be incorporated. Fourthly, temporal confounders such as external policy changes, economic shifts, or trends in the global pharmaceutical market may have influenced the negotiation dynamics observed in this study. These external factors were not explicitly modeled, but they could partially explain trends like the increasing agreement rate over time. For instance, changes in global pharmaceutical market trends or the introduction of supplementary reimbursement policies could have indirectly influenced negotiation dynamics. Recognising such temporal factors can provide a more comprehensive understanding of the observed trends and their implications for policy. Future studies should explore the temporal dynamics of these policies in greater detail to provide a more nuanced interpretation of the results. Therefore, detailed interpretations could only be made based on investigated variables and their results, as detailed information was not publicly available. Lastly, as observed in regression analysis results, rare diseases and cancer treatments generally exerted a negative impact on negotiation agreement rates. However, the 2015 Waiver System showed a positive effect, suggesting its potential to enhance patient access while ensuring financial sustainability. To enhance international applicability, policy recommendations include refining waiver eligibility criteria, strengthening regulatory oversight, and improving transparency in drug pricing processes. These adjustments could address clinical and financial uncertainties, offering valuable insights for other nations considering similar systems. Additionally, tailoring the system to account for variations in regional and global market conditions could enhance its adaptability and scalability.

## 5 Conclusions

This study analysed trends in drug pricing negotiations in Korea from 2007 to 2022, empirically confirming the positive effects of the two‐waiver system introduced in 2015 on negotiation outcomes and patient access. The analysis revealed an overall increase in negotiation agreement rates, particularly for orphan and cancer drugs, driven by targeted institutional interventions and differentiated product characteristics. It was observed that the introduction of the two‐waiver system in 2015 had a positive impact on negotiation agreement rates for rare diseases and cancer treatments. These findings underscore the need for efficient operation of the system to enhance patient accessibility and rationalise financial expenditures. For nations aiming to improve equitable access to high‐cost therapies while maintaining fiscal responsibility, adopting similar frameworks with localised adaptations could provide valuable solutions. Future research and international collaboration are essential to refine and disseminate such systems globally.

## Conflicts of Interest

The author declares no conflicts of interest.

## Data Availability

The data that support the findings of this study are available from the corresponding author upon reasonable request.
